# A hybrid self-supervised model predicting life satisfaction in South Korea

**DOI:** 10.3389/fpubh.2024.1445864

**Published:** 2024-10-17

**Authors:** Hung Viet Nguyen, Haewon Byeon

**Affiliations:** Department of Digital Anti-Aging Healthcare (BK21), Inje University, Gimhae, Republic of Korea

**Keywords:** explainable AI, hybrid model, life satisfaction, self-supervised, TabNet

## Abstract

**Objective:**

Life satisfaction pertains to an individual’s subjective evaluation of their life quality, grounded in their personal criteria. It stands as a crucial cognitive aspect of subjective wellbeing, offering a reliable gauge of a person’s comprehensive wellbeing status. In this research, our objective is to develop a hybrid self-supervised model tailored for predicting individuals’ life satisfaction in South Korea.

**Methods:**

We employed the Busan Metropolitan City Social Survey Data in 2021, a comprehensive dataset compiled by the Big Data Statistics Division of Busan Metropolitan City. After preprocessing, our analysis focused on a total of 32,390 individuals with 51 variables. We developed the self-supervised pre-training TabNet model as a key component of this study. In addition, we integrated the proposed model with the Local Interpretable Model-agnostic Explanation (LIME) technique to enhance the ease and intuitiveness of interpreting local model behavior.

**Results:**

The performance of our advanced model surpassed conventional tree-based ML models, registering an AUC of 0.7778 for the training set and 0.7757 for the test set. Furthermore, our integrated model simplifies and clarifies the interpretation of local model actions, effectively navigating past the intricate nuances of TabNet’s standard explanatory mechanisms.

**Conclusion:**

Our proposed model offers a transparent understanding of AI decisions, making it a valuable tool for professionals in the social sciences and psychology, even if they lack expertise in data analytics.

## 1 Introduction

Life satisfaction pertains to an individual’s subjective evaluation of their life quality, grounded in their personal criteria. This comprehensive evaluation covers multiple areas such as family life, career, and social interactions (1). It stands as an essential cognitive component of subjective wellbeing, offering a reliable gauge of a person’s comprehensive wellbeing status (2). Notably, it exerts a significant influence on various facets of an individual’s mental wellbeing (2). A study conducted by Lewis et al. (3) has demonstrated an inverse relationship between life satisfaction and depressed symptoms, with enhancements in life satisfaction yielding a reduction in such symptoms. Similarly, Fergusson et al. (4) have documented the consequential role of life satisfaction in shaping an individual’s mental wellbeing. Beyond its individual-level implications, life satisfaction also serves as a pivotal metric for assessing the quality of life within a given society (2). The research by Wong et al. (5) based on empirical data, has underscored the differential impacts of social policies on individuals’ life satisfaction. Consequently, the development of an effective predictive model for assessing life satisfaction among individuals carries significant relevance in the realms of mental health research and practice, as well as in formulating policies aimed at enhancing the wellbeing of the broader public.

In the past, numerous scholars have undertaken extensive investigations into the determinants of individuals’ life satisfaction. However, most of these studies have relied upon conventional statistical techniques like regression or mediation analysis, often incorporating control variables in their analyses (6–11). These methods often rely on simplified relationships between variables and may lack the predictive power required to model the complexity of life satisfaction outcome. In contrast, contemporary advancements in computational methodologies, particularly machine learning (ML) and deep learning (DL), present new opportunities for enhancing our understanding of mental health outcomes on an individual basis (12). More recent studies (2, 13) have employed ML models to predict life satisfaction; nonetheless, the application of DL models, capable of revealing intricate patterns within large datasets, is yet inadequately investigated in this domain. Moreover, a notable constraint of deep learning models is their “black box” characteristic, which complicates the interpretation of results and the comprehension of the underlying causes influencing predictions.

To address those challenges, our research seeks to answer these three key research questions:

- Question 1: How effectively can a DL model, specifically the self-supervised pretraining TabNet (SSP-TabNet) model (14), predict life satisfaction from large-scale social survey data compared to traditional supervised ML models?
- Question 2: What are the most significant predictors of life satisfaction, and how can we interpret the model’s decision-making process in a manner that is accessible to non-technical users?
- Question 3: How does the integration of the Local Interpretable Model-agnostic Explanation (LIME) (15) interpretability framework enhance the explanation of local model behaviors, particularly in the context of predicting life satisfaction?

In the context of the rapidly evolving field of artificial intelligence, recent advancements have highlighted the efficacy of self-supervised learning in acquiring valuable data representations (16). Notably, this success has primarily been evident in data modalities like images (17, 18), audio (19), and text (20, 21). This achievement hinges on the exploitation of inherent spatial, temporal, or semantic structures within the data (22, 23). However, when it comes to tabular datasets, frequently employed in domains such as healthcare, such structural characteristics are often limited or absent. In recent, Arik and Pfister (14) presented a groundbreaking DL architecture known as the TabNet model, specifically designed for handling tabular data. In addition to its supervised architecture, TabNet was the first use of self-supervised pre-training technique to tabular data, resulting in significant performance improvements. The TabNet model has laid the foundation for further exploration and advancements in leveraging self-supervised learning technique within tabular data applications, particularly in vital fields like healthcare.

In this research endeavor, our primary objective is to develop a self-supervised pre-training methodology tailored for the prediction of individuals’ life satisfaction in the South Korean context. We employed the SSP-TabNet model as a key component of this approach. A notable advantage of SSP-TabNet is that it eliminates the need for feature selection, a critical step in traditional supervised machine learning models. Additionally, SSP-TabNet provides strong interpretability, enabling both localized and global insights into the decision-making process. Nonetheless, the localized interpretability of TabNet, reliant on access to its decision masks, is not easy to understand for professionals within fields such as social work and psychology, who may not possess advanced proficiency in data analysis techniques (24). To address this issue, we have integrated the SSP-TabNet model with the Local Interpretable Model-agnostic Explanation (LIME) (15) technique. This hybrid model enhances the ease and intuitiveness of interpreting local model behavior, surpassing the inherent complexities of SSP-TabNet’s native interpretation features, thus making it more accessible to a broader audience.

## 2 Materials and methods

### 2.1 Dataset

In our research, we employed the Busan Metropolitan City Social Survey Data in 2021, a comprehensive dataset compiled by the Big Data Statistics Division of Busan Metropolitan City. This dataset provides a comprehensive understanding of the living conditions and civic engagement levels of the city’s residents. It is specifically designed to assess the quality of civic life and overall welfare, forming the foundational data for policymaking and community development initiatives in Busan. The survey protocol received ethical approval (IRB approval no. 17339) from Statistics Korea, ensuring adherence to ethical research standards.

The survey encompassed all residents aged 15 years and above within the geographic boundaries of Busan Metropolitan City, with the sampling frame derived from the 2019 Population and Housing Census - a nationwide survey. Employing a probability proportional systematic sampling approach, the survey drew a final sample of 17,860 households (comprising 940 survey districts, with 19 households per district) residing in Busan Metropolitan City at the time of the survey administration. Our analysis, subsequently, focused on a total of 32,390 individuals who successfully completed the survey.

### 2.2 Data preprocessing

**Figure 1:**
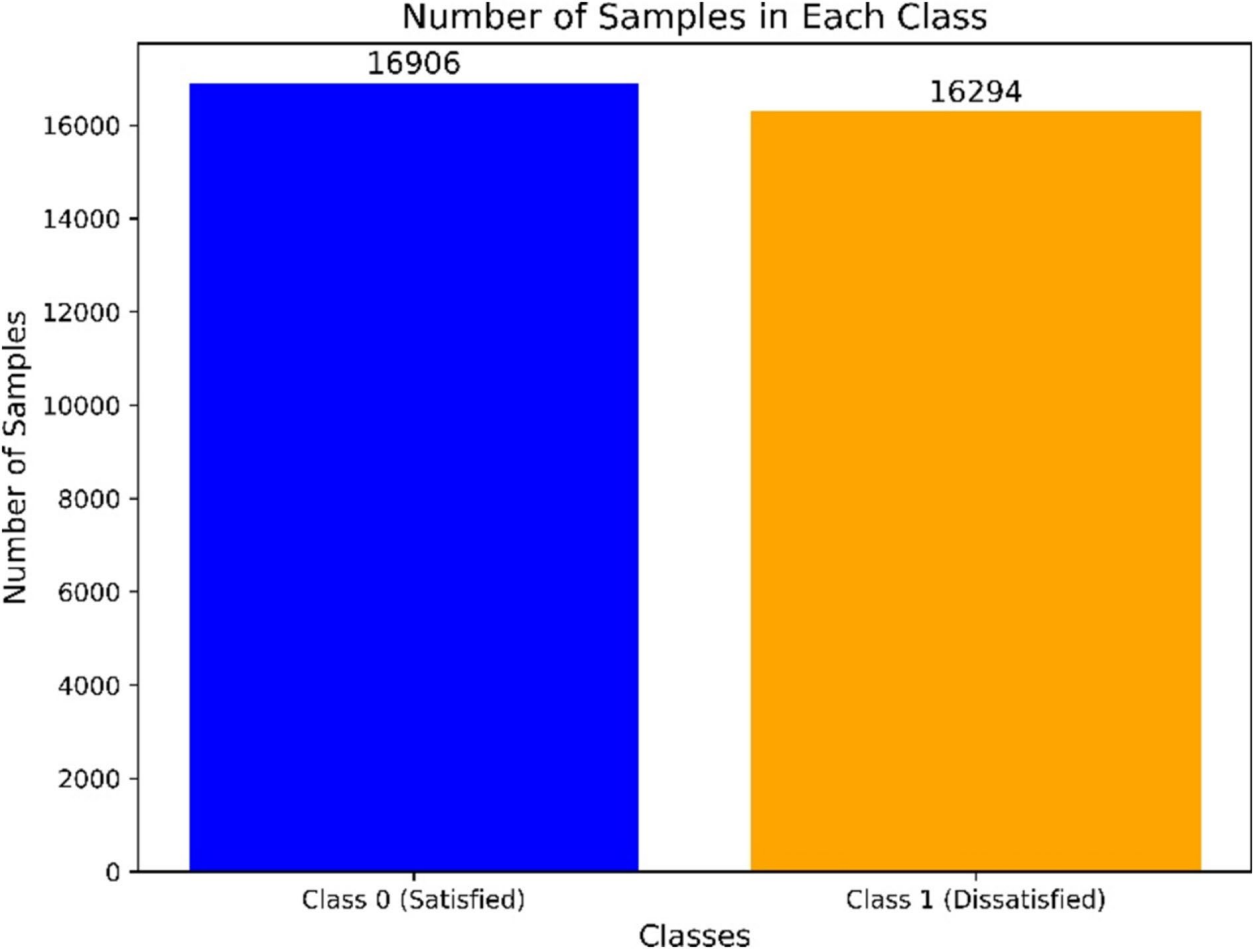
Target distribution.

The original dataset consisted of 32,390 samples with 132 features. To prepare the dataset for model training, we applied several preprocessing steps, which are outlined below:

1. Removal of redundant columns: we first eliminated two columns containing superfluous serial number information, as they had no relevance to the analysis.
2. Handling missing data: given that the survey allowed for optional responses, some columns contained a high percentage of missing data. We adopted a threshold-based approach to exclude columns with a significant proportion of missing values. Specifically, any features where more than 50% of the values were missing were removed. This decision was based on the assumption that such features would not provide reliable insights or sufficient information for model training. The excluded features were primarily non-critical or supplementary variables, which were not essential for achieving our research objectives. After removing columns with more than 50% missing values, the dataset included 32,390 samples and 51 variables, including the target feature. A detailed overview of these 51 variables is provided in Supplementary Table S1.
3. Target feature definition: the target feature for this study was defined as “life satisfaction during the COVID-19 pandemic,” measured on a 10-point scale from 1 (least satisfied) to 10 (most satisfied). To simplify the classification task, we recategorized the target feature into two classes: scores of 5 or below were classified as “dissatisfied” (class 1), and scores of 6 or above were classified as “satisfied” (class 0). This recategorization yielded 16,906 samples in class 0 (satisfied) and 16,294 samples in class 1 (dissatisfied), as illustrated in Figure 1.
4. Data splitting: the dataset was randomly stratified into 80% for training (25,912 samples) and 20% for testing (6,478 samples), ensuring that both classes were equally represented in each set. The training set facilitated model development and hyperparameter optimization, while the test set assessed the model’s efficacy on unseen data.

In our study, after removing columns with more than 50% missing values, we did not apply any additional feature selection methods to further diminish the dimensionality of the variables. The reason is because SSP-TabNet’s architecture inherently performs automatic feature selection through its Mask layer, unlike conventional machine learning models, which often require a manual feature selection process. The Mask layer in SSP-TabNet identifies the most relevant features for each decision step during training, thereby eliminating the need for manual feature selection.

### 2.3 Development of self-supervised

TabNet, introduced by Arik et al. (14), builds upon the end-to-end retraining and representation learning feature characteristics inherent to deep neural networks (DNN). Notably, TabNet combines these traits with the interpretability and sparse feature selection capabilities often associated with tree-based models. In the realm of real-world dataset analysis, TabNet was demonstrated that outperformed traditional ML algorithms, highlighting its proficiency in delivering enhanced accuracy. Another notable advantage of TabNet is its elimination of the need for feature selection process by its Mask layer. Furthermore, TabNet provides interpretability by identifying the most critical attributes for each sample. This attribute selection process aids in understanding the model’s decision-making rationale. In recent studies, TabNet has found applications across diverse domains, including healthcare (24), fraud detection (25), and energy management (26), showcasing its versatility and utility in a range of practical contexts.

The self-supervised pretraining TabNet (SSP-TabNet) architecture comprises two essential elements: a TabNet encoder and a TabNet decoder module. Central to this self-supervised learning approach is the acknowledgment of the intrinsic interconnections among various features within a single data sample (16). The approach initiates with the strategic masking of certain features. Subsequently, the encoder-decoder framework is utilized to predict these concealed features. This process effectively equips the TabNet encoder module with the capacity to aptly characterize the distinctive features of each sample. This approach accelerates model convergence and augments overall model performance, enhancing its capacity to identify complex relationships within the data. In summary, we selected SSP-TabNet as the primary model due to its ability to handle tabular data effectively, its automatic feature selection capabilities, and its interpretability, which are particularly important in the context of social survey data.

#### 2.3.1 TabNet encoder structure

**Figure 2:**
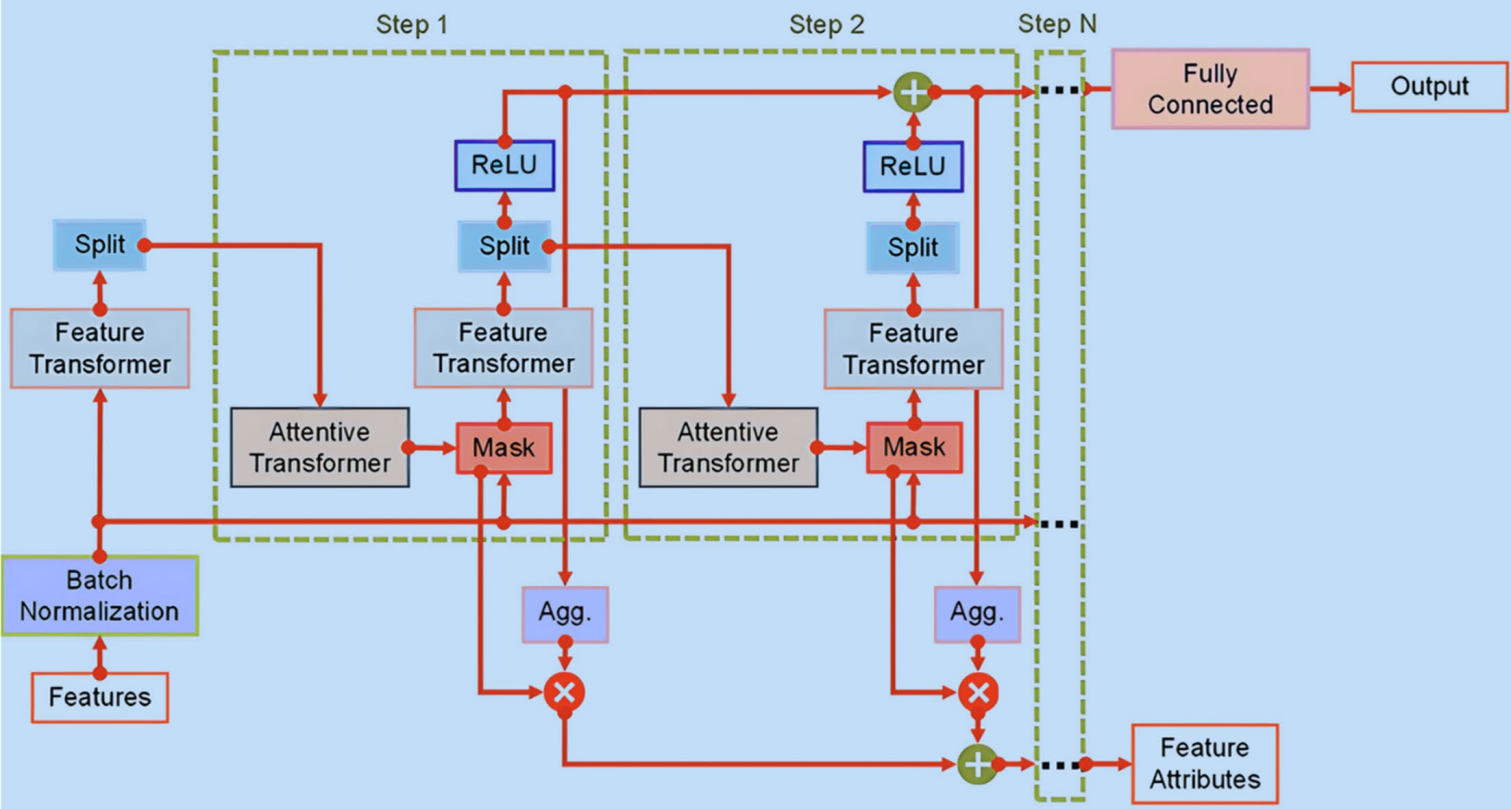
TabNet encoder structure.

The TabNet encoder structure, as illustrated in Figure 2, is composed of sequential multi-steps (N_steps_). At each step $i^{th}$ , it takes in refined data from the previous stage, denoted as the ${\left(i-1\right)}^{th}$ , to determines the relevant features to employ. Subsequently, it generates processed feature representations, which are collectively integrated to inform the overall decision-making process. The model is designed to process datasets with a defined batch size (B) and features of D-dimensions, and it operates independently of global feature normalization. Prior to entering the Feature Transformer, the data is subject to batch normalization (BN).

**Figure 3:**
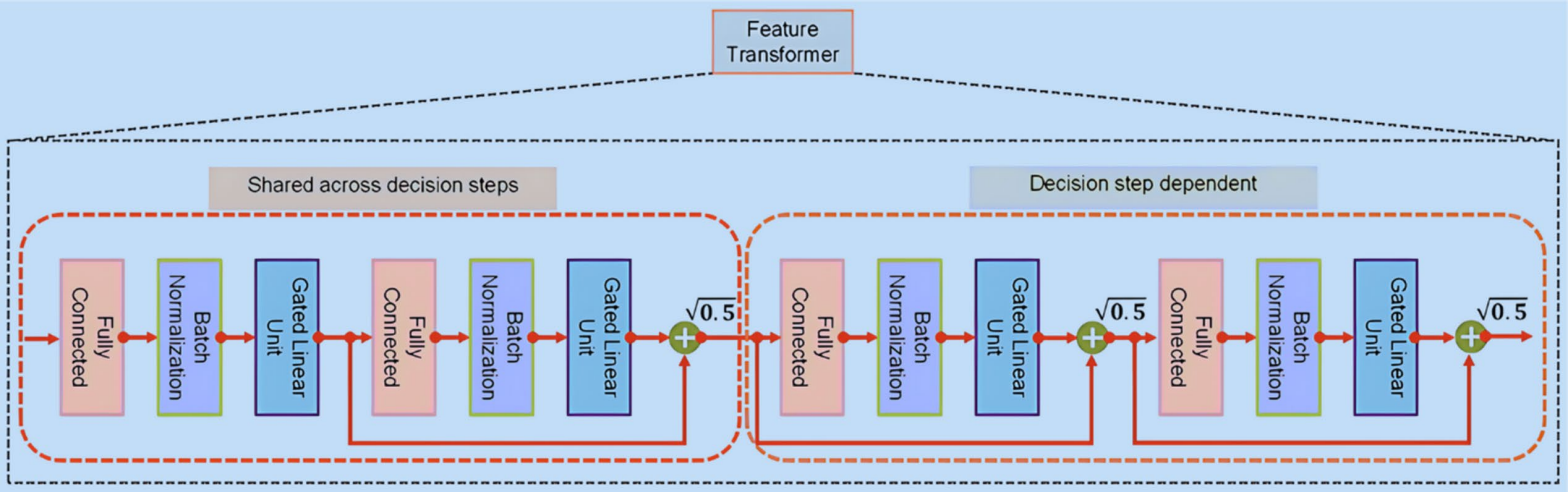
Feature transformer.

The Feature Transformer, illustrated in Figure 3, encompasses a series of n distinct gated linear unit (GLU) blocks. Within each Feature Transformer block comprises three key layers: fully connected (FC), BN, and GLU. In configurations utilizing four GLU blocks, two are designed to function in tandem, while the remaining two operate independently. This design promotes efficient learning. Notably, a skip connection links consecutive blocks, and a normalization step with a factor of $\sqrt{0.5}$ follows each block to ensure stability, preventing significant variance fluctuations (27). Upon processing the features that have undergone batch normalization, the Feature Transformer conveys this refined information to the Attentive Transformer at the $i^{th}$ step via a split layer.

**Figure 4:**
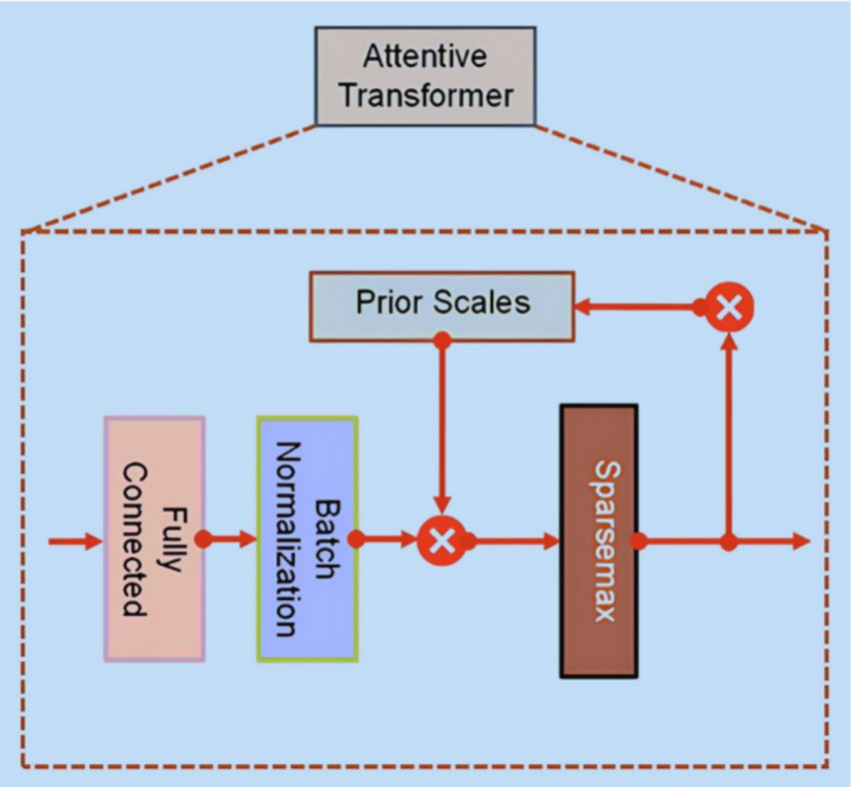
Attentive transformer.

The Attentive Transformer (Figure 4) consists of four crucial layers: FC, BN, Prior Scales, and Sparsemax. The input sourced from the split layer is first processed via the FC and BN layers. Subsequently, it is directed to the Prior Scales layer, where the importance of features relevant to the present decision-making step is aggregated, as described in Equation 1:


(1) $$P\left[i\right]=\overset{i}{\underset{j=1}{\prod }}\left(\gamma -M\left[j\right]\right)$$

where γ is the relaxation parameter.

The primary role of an Attentive Transformer is to compute the Mask layer for the current step, building upon the results from the preceding step. This learnable Mask facilitates the sparse selection of the most critical features, thus improving the model’s efficiency in terms of parameter utilization. By focusing the learning capabilities of each decision step on relevant features, this method minimizes the resources expended on non-essential elements. Importantly, the Attentive Transformer plays a crucial role in deriving masks from the processed features obtained in the preceding step, due to the multiplicative nature of the masking procedure, as demonstrated in Equation 2:


(2) $$M\left[i\right]=\mathrm{sparsemax}\left(P\left[i-1\right]\right)*h_{i}\left(a\left[i-1\right]\right)$$

where $h_{i}$ represents the trainable function used to represent the FC and BN layers, $P\left[i-1\right]$ denotes the preceding scales item, and the sparsemax layer is employed for coefficient normalizing leading to sparse feature selection.

The Mask layer is subsequently directed to the Feature Transformer, where an analysis of the filtered features occurs. Within this transformation process, the data is segregated into two distinct outputs, as presented in Equation 3:


(3) $$\left[d\left[i\right],a\left[i\right]\right]=f_{i}\left(M\left[i\right]*f\right)$$

where $a\left[i\right]\in {\mathbb{R}}^{B*N_{a}}$ represents the data utilized in the subsequent stage of the Attentive Transformer, and $d\left[i\right]\in {\mathbb{R}}^{B*N_{d}}$ denotes the outcome generated by the decision step.

Drawing inspiration from the concept of aggregating tree models, the output vectors $d\left[i\right]$ generated from all the decision steps were consolidated into a unified vector, represented as $d_{\mathrm{out}}$ . Subsequently, a FC layer is employed to map this final aggregated output. This aggregation process is defined as shown in Equation 4:


(4) $$d_{\mathrm{out}}=\overset{N_{\mathrm{steps}}}{\underset{i=1}{\sum }}\mathrm{ReLU}\left(d\left[i\right]\right)$$

#### 2.3.2 TabNet decoder structure

**Figure 5:**
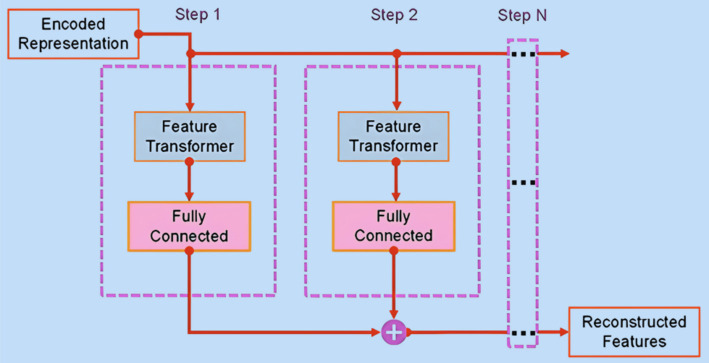
TabNet decoder structure.

To fulfill the self-supervised learning objective of TabNet model, an accompanying decoder architecture (Figure 5) has been introduced to facilitate the reconstruction of masked features from the encoded phase. In this reconstruction process, a binary mask, labeled as S (with $S\in {\left\{0,1\right\}}^{B\times D}$ ), is used. Within this framework, both the FC layer and Feature Transformer layer work collaboratively to predict the masked feature at each step. This prediction is guided by the minimization of the reconstruction loss, as expressed in Equation 5:


(5) $$\overset{B}{\underset{b=1}{\sum }}\overset{D}{\underset{j=1}{\sum }}|\frac{\left({\hat{f}}_{b,j}-f_{b,j}\right).S_{b,j}}{\sqrt{\sum_{b=1}^{B}{\left(f_{b,j}-1/B\sum_{b=1}^{B}f_{b,j}\right)}^{2}}}|$$

where ${\hat{f}}_{b,j}$ and $f_{b,j}$ respectively represent the expected feature importance score and the feature importance score attributed to the *j^th^* feature within the *b^th^* sample.

In our study, we harnessed the pytorch_tabnet package version 4.1 to craft the self-supervised pre-training TabNet (SSP-TabNet) model. The overarching objective of the self-supervised pre-training phase involves TabNet mapping the embedding space of unlabeled data, from which we derived pretrained weights. This process entails the utilization of the TabNetPretrainer to acquire knowledge from the unlabeled dataset. Subsequently, we transitioned to a fine-tuning stage, where these pretrained weights were employed to train a TabNetClassifier. This classifier was further trained using labeled data to enhance its predictive capabilities. For the optimization of hyperparameters in both the pretraining and fine-tuning phases, we leveraged the Optuna (28) framework. The fine-tuned hyperparameters are meticulously detailed in Table 1. Ultimately, the optimized model is employed to evaluate performance scores through a 5-fold cross-validation procedure. To facilitate a performance comparison between the SSP-TabNet model and the standard TabNet model, we adopted a consistent approach by applying the same hyperparameters used for SSP-TabNet to the standard supervised TabNet model. However, it is worth noting that the “from_unsupervised” hyperparameter was not utilized when fitting the normal TabNet model.

**Table 1:** Hyperparameters when developing SSP-TabNet optimized by Optuna.

Model	Hyperparamters
pretrain	TabNetPretrainer: n_a = 40, n_d = 40, n_steps = 4, momentum = 0.12, lamda_sparse = 2.96e-05, optimizer_params = {‘lr’: 0.03}, optimizer_fn = torch.optim.adam.Adam, scheduler_params = {‘patience’: 5, ‘mode’: ‘min’, ‘factor’: 0.9, ‘min_lr’: 1e-05}, scheduler_fn = torch.optim.lr_scheduler.ReduceLROnPlateau, gamma = 1.9, mask_type = ‘entmax’, batch_size = 512, virtual_batch_size = 256, pretraining_ratio = 0.6, max_epochs = 500, patience = 30
SSP-TabNet	TabNetClassifier: n_a = 32, n_d = 32, n_steps = 9, momentum = 0.26, lamda_sparse = 6.39e-05, optimizer_params = {‘lr’: 0.009}, mask_type = ‘sparsemax’, optimizer_fn = torch.optim.adam.Adam, gamma = 1, virtual_batch_size = 128, batch_size = 1,024, eval_metric = [‘auc’], max_epochs = 500, patience = 30, from_upsupervised = pretrain
TabNet	TabNetClassifier: n_a = 32, n_d = 32, n_steps = 9, momentum = 0.26, lamda_sparse = 6.39e-05, optimizer_params = {‘lr’: 0.009}, mask_type = ‘sparsemax’, optimizer_fn = torch.optim.adam.Adam, gamma = 1, virtual_batch_size = 128, batch_size = 1,024, eval_metric = [‘auc’], max_epochs = 500, patience = 30

Unsupervised pretraining = pretrain, Self-supervised pretraining TabNet Classification model = SSP-TabNet, TabNet Classification model = TabNet.

### 2.4 Performance evaluation method

#### 2.4.1 Comparing performance to ML models

While Arik et al. (14) demonstrated TabNet’s superiority over traditional ML algorithms, recent studies (29–31) have continued to show instances where tree-based models still outperform DL when dealing with tabular data. This is due to tree-based models’ ability to effectively handle high-dimensional data and perform well on structured datasets. Therefore, in our investigation, we opted to include a variety of tree-based models, specifically CatBoost (32), LightGBM (33), XGBoost (34), Gradient Boosting (GBC) (35), Extra Tree (ET) (36), Random Forest (RF) (37), and Decision Tree (DT) (38) to conduct a performance comparison with the SSP-TabNet model. Including these models ensures a fair and comprehensive assessment of SSP-TabNet’s performance. Additionally, to guarantee a fair evaluation, we also fine-tuned the hyperparameters of these tree-based models using the Optuna framework. These models’ hyperparameters were outlined in Table 2.

**Table 2:** Hyperparameters of tree-based models.

Model	Hyperparamters
CatBoost	CatBoostClassifier: depth = 10, l2_leaf_reg = 200, border_count = 254, n_estimators = 300, eta = 0.14
LightGBM	LGBMClassifier: max_depth = −1, learning_rate = 0.04, min_child_weight = 0.001, min_child_samples = 4, reg_alpha = 1.77e-09, reg_lambda = 0.02, bagging_freq = 2, feature_fraction = 0.4, bagging_fraction = 0.93, num_leaves = 256, n_estimators = 300
XGBoost	XGBClassifier: learning_rate = 0.07, max_depth = 11, colsample_bytree = 0.64, reg_alpha = 6.07e-06, reg_lambda = 10, subsample = 0.88, objective = ‘binary:logistic’, n_estimators = 300
GBM	GradientBoostingClassifier: max_features = 0.4, max_depth = 11, min_samples_leaf = 5, min_samples_split = 2, min_impurity_decrease = 1e-09, subsample = 0.86, n_estimators = 300, learning_rate = 0.05
ET	ExtraTreesClassifier: min_samples_split = 5, min_samples_leaf = 3, criterion = ‘entropy’, n_estimators = 150, max_depth = 20
RF	RandomForestClassifier: criterion = ‘entropy’, n_estimators = 120, max_depth = 25, min_samples_leaf = 4, min_samples_split = 6, min_impurity_decrease = 0.002, bootstrap = True
DT	DecisionTreeClassifier: min_samples_leaf = 2, min_impurity_decrease = 0.001, min_samples_split = 10, max_depth = 7

CatBoost Classification model = CatBoost, LightGBM Classification model = LightGBM, XGBoost Classification model = XGBoost, Gradient Boosting Classification model = GBM, Extra Tree Classification model = ET, Random Forest Classification mode = RF, Decision Trees Classification model = DT.

#### 2.4.2 Performance metrics

In binary classification problems, the most commonly employed evaluation metrics encompass accuracy, recall, precision and the F1-score. These metrics collectively provide a statistical assessment of a classifier model’s performance. The formulas for these metrics are presented in Equations 6–9:


(6) $$\mathrm{Accuracy}=\frac{True_{\mathrm{possitive}}+True_{\mathrm{negative}}}{\mathrm{Total}\;\mathrm{predictions}}$$

(7) $$\mathrm{Recall}=\frac{True_{\mathrm{possitive}}}{True_{\mathrm{positive}}+\mathrm{Fals}e_{\mathrm{negative}}}$$

(8) $$\mathrm{Precision}=\frac{True_{\mathrm{possitive}}}{True_{\mathrm{positive}}+\mathrm{Fals}e_{\mathrm{positive}}}$$

(9) $$F1-\mathrm{score}=2\times \frac{\mathrm{Recall}\times \mathrm{Precision}}{\mathrm{Recall}+\mathrm{Precision}}$$

where $True_{\mathrm{negative}}$ and $True_{\mathrm{possitive}}$ signify the accurate predictions made for the satisfied (class 0) and dissatisfied (class 1) respectively; while $\mathrm{Fals}e_{\mathrm{negative}}$ and $\mathrm{Fals}e_{\mathrm{positive}}$ represent the erroneous predictions of the class 0 and the class 1, respectively.

Furthermore, the “Area under the Receiver Operating Characteristic Curve” (AUC) was also employed to assess the performance of models. The AUC score is calculated as shown in Equation 10:


(10) $$AUC=\overset{0}{\underset{1}{\int }}TPR\left(t_{i}\right)\;d\left(FPR\left(t_{i}\right)\right)$$

where $FPR\left(t_{i}\right)$ and $TPR\left(t_{i}\right)$ are “false positive rate” and “true positive rate” for a threshold $t_{i}$ . In our study, we operated under the assumption that a model achieving the highest AUC exhibited the most robust predictive capability. In cases where multiple models displayed similar AUC values, we prioritized the model with the highest accuracy as the superior choice.

In this study, we conducted all our analyses using Python 3.10.13 (https://www.python.org, accessed on September 29, 2023). To rigorously evaluate the performance metrics across all models, we adopted the stratified 5-fold cross-validation technique. This method entails partitioning the dataset into k equally sized segments, with instances randomly distributed across these segments. Stratified cross-validation’s distinctive aspect is its ability to preserve a distribution of class labels in each segment that mirrors the original dataset’s distribution. In the k iterations of evaluating the model, each segment is utilized once as the validation set, while the others form the training data. This approach, by testing the model on various data subsets, provides a more comprehensive assessment of its generalization ability, thereby strengthening the credibility of our findings.

### 2.5 Local interpretable model-agnostic explanations (LIME)

Local Interpretable Model-Agnostic Explanations (LIME) is a widely adopted model-agnostic technique employed to elucidate the inner workings of black-box models. Its fundamental approach involves creating localized interpretable models centered around a specific data instance, thereby approximating how the black-box model behaves in that particular context. LIME was originally developed by Ribeiro et al. (15) and has gained significant popularity for explaining the predictions made by complex ML models. Notably, researchers have harnessed LIME for various applications. For instance, Nguyen et al. (39) applied LIME to predict depression in Parkinson’s Disease patients. Mardaoui et al. (40) employed LIME to interpret text data effectively. Mizanur et al. (41) used LIME to explain for loan approval prediction model. Additionally, Jain et al. (42) utilized LIME to offer insights into sentiment analysis results derived from social media texts. These studies collectively demonstrate LIME’s utility in providing profound insights into the predictions of black-box models across diverse domains.

In order to provide an explanation for a particular observation, LIME operates by repeatedly perturbing an observation to create a set of replicated feature data. This perturbed data is then subjected to predictions using a model, such as TabNet. Each point in the perturbed dataset is compared with the original data point, and the Euclidean distance between them is calculated. This distance provides an indication of how much the perturbed data point diverges from the original observation. This metric is crucial in identifying which input features are considered significant by the model for its predictions. The ultimate objective of LIME is to create an explainer that is both dependable and interpretable. To achieve this goal, LIME focuses on minimizing the following objective function (Equation 11):


(11) $$\xi \left(x\right)=\mathrm{argmi}n_{g\in G}\;L\left(f,g,\pi_{x}\right)+\Omega \left(g\right)$$

where *f* represents the original model, *g* denotes the interpretable model, *x* represents the original observation, $\pi_{x}$ signifies the proximity measure computed across all permutations to the original observation. In addition, $L\left(f,g,\pi_{x}\right)$ serves as a metric assessing the extent to which the interpretable model *g* faithfully approximates the behavior of the original model *f* within the locality defined by *π*. Lastly, $\Omega \left(g\right)$ represents a measure of model complexity. In the context of our study, we selected a specific instance for analysis to illustrate how the LIME model collaborates with the SSP-TabNet model to predict an individual’s life dissatisfaction outcome. This focused examination allowed us to showcase the practical application of LIME in tandem with the SSP-TabNet model for predictive purposes.

## 3 Results

### 3.1 Performance comparisons results

Table 3 presents a summary of the predictive performance of 9 optimized models on the training set. The SSP-TabNet model showed the highest performance, achieving an AUC score of 0.7778, significantly higher than the other models. Models such as CatBoost, XGBoost, LightGBM, RF, and GBC followed, with AUC scores of 0.7735, 0.7689, 0.7683, 0.7636, and 0.7598, respectively. Conversely, the ET, DT, and TabNet models exhibited the lowest AUC scores among the nine models, each attaining an AUC value of 0.7521.

**Table 3:** Performance comparison of optimized models on the training set.

Model	AUC	Accuracy	Precision	Recall	F1-score
SSP-TabNet	0.7778	0.7047	0.7066	0.7064	0.7065
CatBoost	0.7735	0.7012	0.6963	0.7057	0.701
XGBoost	0.7689	0.6982	0.6924	0.7031	0.6977
LightGBM	0.7683	0.6979	0.6994	0.7	0.6997
RF	0.7636	0.6945	0.6959	0.6965	0.6962
GBC	0.7598	0.6872	0.6914	0.6883	0.6898
ET	0.7521	0.6827	0.6928	0.6817	0.6872
DT	0.7321	0.6719	0.6709	0.6749	0.6729
TabNet	0.7321	0.6722	0.6703	0.6756	0.6729

Self-supervised pretraining TabNet Classification model = SSP-TabNet, TabNet Classification model = TabNet, LightGBM Classification model = LightGBM, XGBoost Classification model = XGBoost, Gradient Boosting Classification model = GBM, CatBoost Classification model = CatBoost, Extra Tree Classification model = ET, Decision Trees Classification model = DT, Random Forest Classification mode = RF.

The SSP-Tabnet model not only excelled in AUC scores but also demonstrated outstanding performance in other evaluation metrics, such as accuracy, precision, recall, and F1-score. For accuracy, SSP-Tabnet obtained the best score at 0.7047, meaning that the model correctly predicted life satisfaction in 70.47% of cases. CatBoost (0.7012), XGBoost (0.6982), LightGBM (0.6979), and RF (0.6945) followed closely behind. Lower accuracies were observed for GBC (0.6872), ET (0.6827), TabNet (0.6722), and DT (0.6719). SSP-TabNet attained precision of 0.7066, recall of 0.7064, and F1-score of 0.7065, reflecting its balanced performance across different evaluation measures.

**Figure 6:**
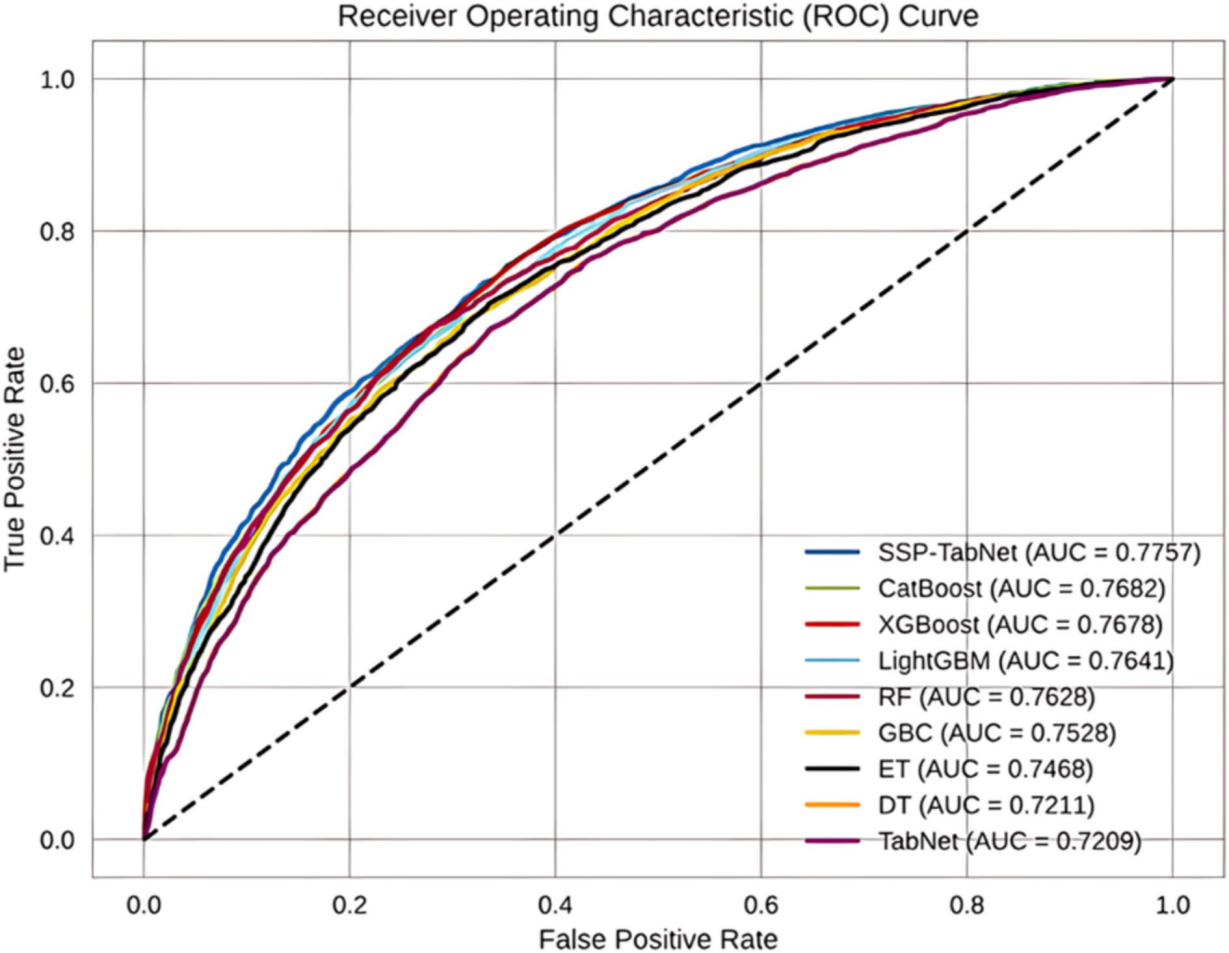
ROC curves of all models evaluated on the test set.

On the test set, the SSP-TabNet model continued to outperform the other models with an AUC score of 0.7757, as depicted in Figure 6. Following in the rankings were CatBoost (0.7682), XGBoost (0.7678), LightGBM (0.7641), RF (0.7628), GBC (0.7468), ET (0.7468), DT (0.7211), and TabNet (0.7209). According to these results, the SSP-TabNet model exhibited outstanding performance relative to all other models examined in this study, particularly in predicting individuals’ life satisfaction, as observed in both the training and test datasets.

### 3.2 Results of global and local interpretability by TabNet

**Figure 7:**
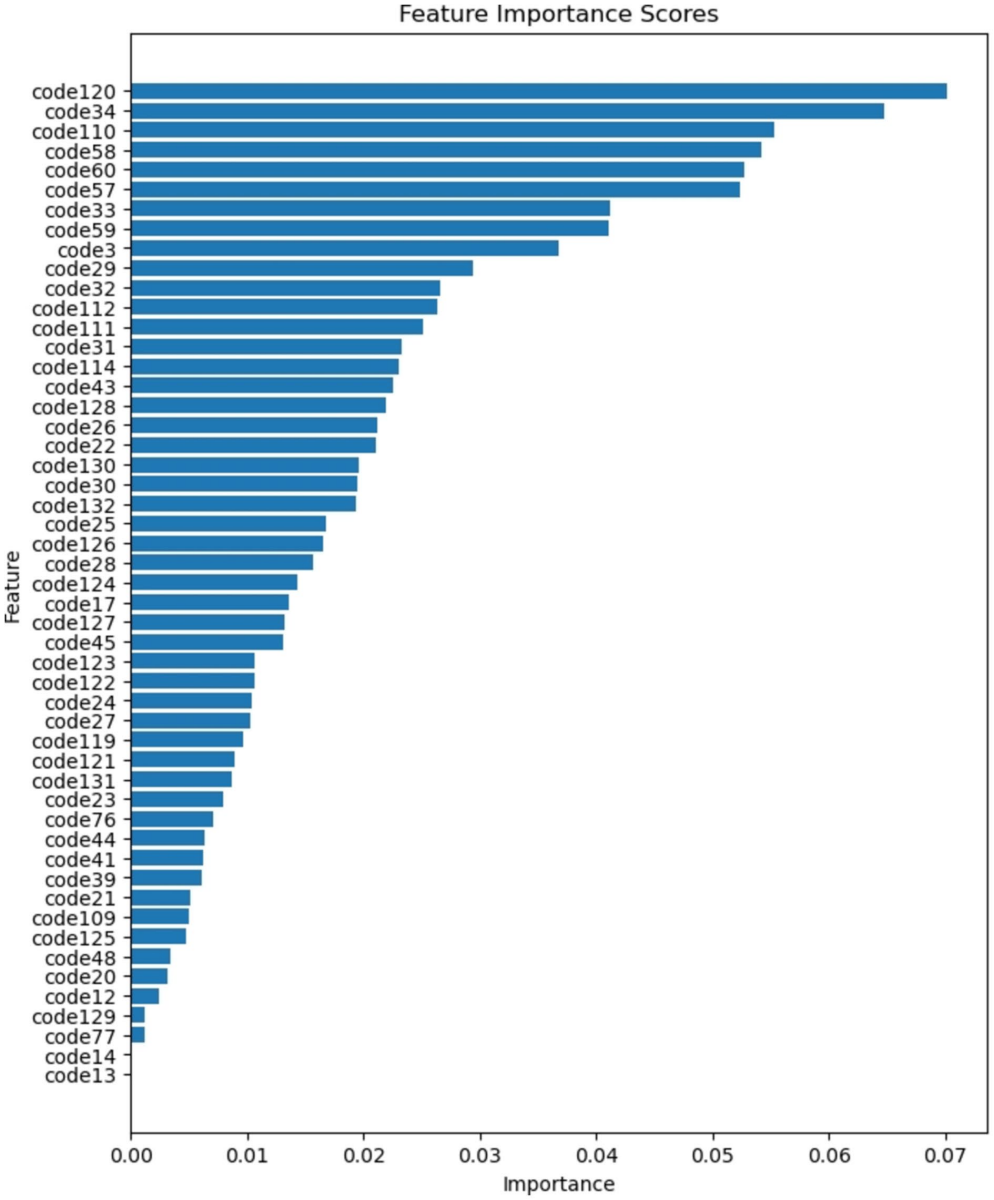
Global feature importance ranking.

Figure 7 provides a visual representation of the global importance attributed to each feature following the training of SSP-TabNet. The analysis reveals that the top five crucial features, ranked by importance, for SSP-TabNet are as follows.

- code120 (Changes in Daily Life Due to COVID-19: Employment activities)
- code34 (Do you commute or go to school?)
- code110 (Age)
- code58 (Effects of School Education: Personality Development)
- code60 (Effects of School Education: Utilization in Daily Life and Employment)

**Figure 8:**
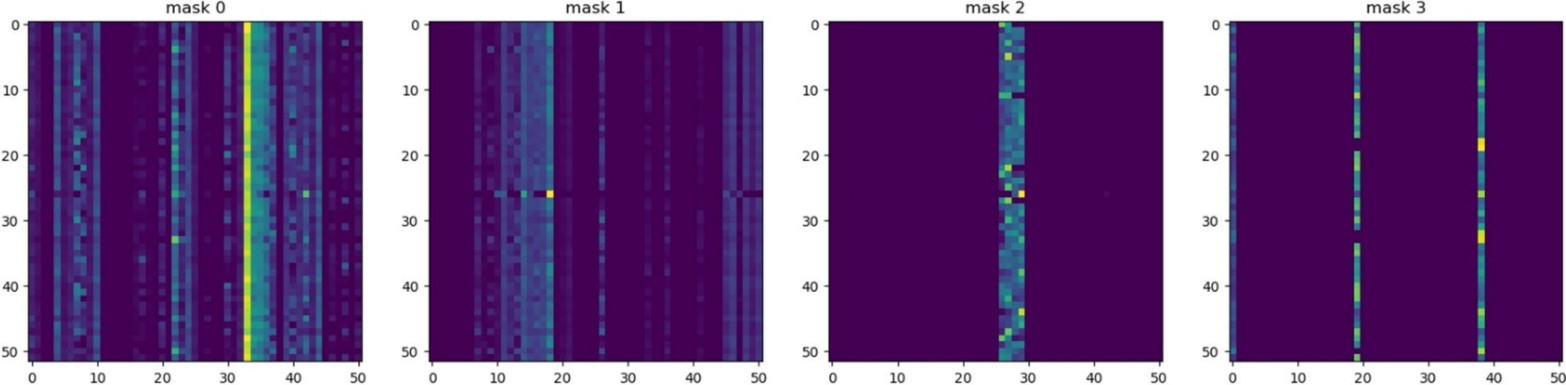
TabNet’s feature importance masks.

In terms of local explanation by SSP-TabNet, Figure 8 illustrates how SSP-TabNet selects and weighs features at different decision steps in the model. The visualization utilizes color intensity to convey the feature weight assigned at each decision step. Brighter colors indicate features that were given more weight in that step, meaning they had a greater impact on the model’s decision at that point. Each decision step assigns different weights to individual features, demonstrating that the model considers each case separately and gives different importance to features depending on the individual data point.

### 3.3 Local interpretation SSP-TabNet with LIME

**Figure 9:**
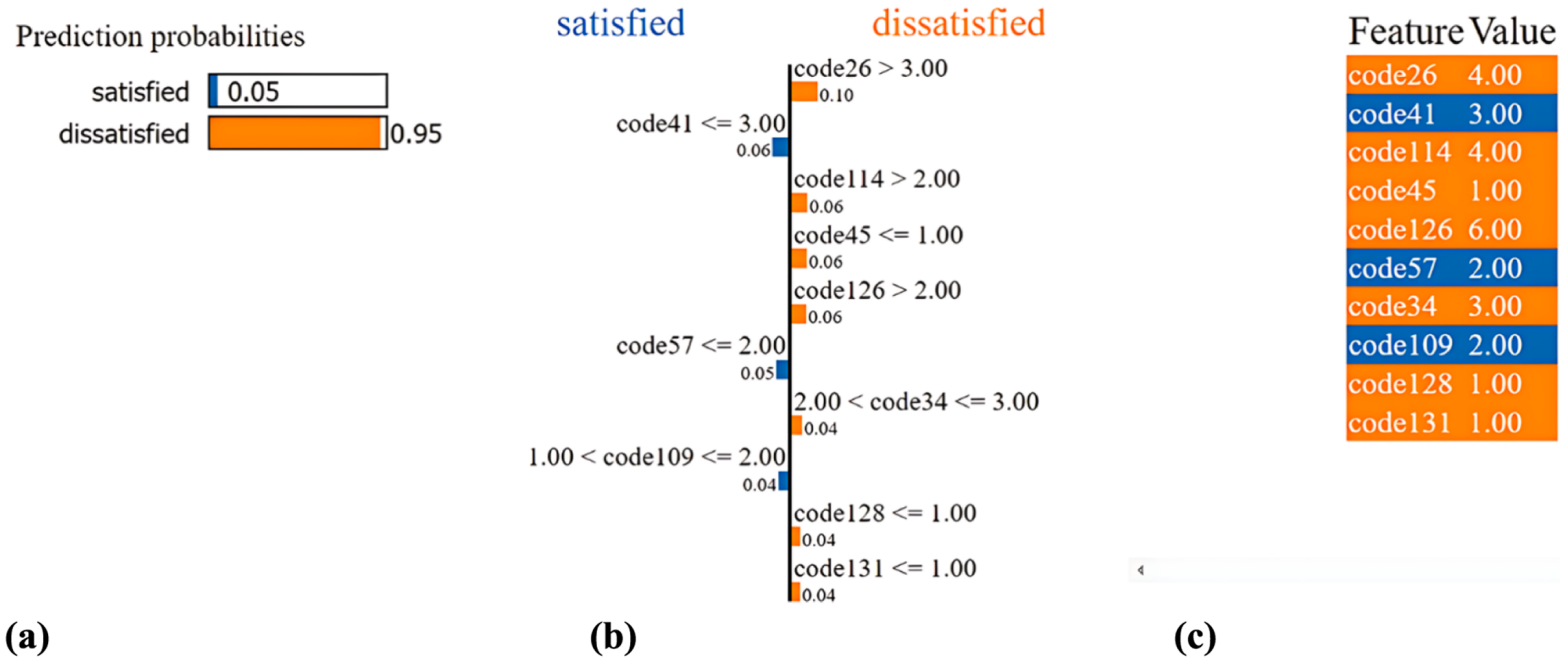
Example of an individual with prediction result as “dissatisfied” for life. **(a)** Prediction probabilities. **(b)** LIME methodology. **(c)** Features’ contribution.

Figure 9 provides a detailed description of an individual for whom the predicted outcome is “dissatisfied” with life. Given the individual’s states and attributes, the SSP-TabNet model predicted a “dissatisfied” rate of 95%, as illustrated in Figure 9a. In Figure 9b visualization, the blue bars symbolize those variables that significantly contribute to negating the prediction, aligning with the “dissatisfied” outcome. Conversely, the orange bars depict variables that support the prediction, correlating with a “satisfied” outcome. According to the explanation provided in Figure 9b, at the time of the prediction, code26 (Residential Environment Satisfaction-Housing) emerged as the most influential factor contributing to the prediction, with a weight of 0.1.

In Figure 9c, we offer a summary of this individual’s state and the contributing circumstances, which are derived from 10 out of the 51 features. This individual’s attributes can be explained as:

- code26: 4 (Residential Environment Satisfaction-Housing: Not quite)
- code41: 3 (Satisfaction with Leisure Activities: Average)
- code114: 4 (Marital Status: Widowed)
- code45: 1 (Weekend and Holiday Leisure Activities: Watching TV)
- code126: 6 (Changes in Daily Life Due to COVID-19 - Gatherings with Family, Friends, and Colleagues: Not applicable)
- code57: 2 (Effects of School Education - Acquisition of Knowledge and Skills: Somewhat)
- code34: 3 (Do you commute or go to school?: Do not commute or go to school)
- code109: 2 (Gender: Female)
- code128: 1 (Behavior Changes for COVID-19 Prevention - Canceling Gatherings and Not Attending Events: Always)
- code131: 1 (Behavior Changes for COVID-19 Prevention - Using Soap and Hand Sanitizer: Always)

## 4 Discussion

This research represents, to our knowledge, the first instance of developing a DL model to predict life satisfaction using tabular data extracted from social survey in general population. In this research, we evaluated the effectiveness of the novel SSP-TabNet model in predicting life satisfaction indices. Notably, our self-supervised pre-training approach showcased superior performance compared to conventional tree-based algorithms, including CatBoost, LightGBM, XGBoost, GBC, ET, RF, and DT, particularly when applied to social survey tabular datasets. Our comparisons revealed that while the standard supervised TabNet model yielded an AUC of 0.7321 on the training set and 0.7209 on the test set; while the SSP-TabNet model resulted in a commendable AUC of 0.7778 and 0.7757 on the respective sets. In addition, the integration of the SSP-TabNet with the LIME interpretability framework offers a transparent understanding of AI decisions, making it a valuable tool for professionals in the social sciences and psychology, even if they lack expertise in data analytics.

In the realm of data science, deciphering TabNet’s mask as illustrated in Figure 8 is straightforward. Yet, for experts in social sciences and psychology, without an in-depth background in data analytics, grasping the intricacies of TabNet’s mask can be challenging (24). While TabNet provides local explanations via feature masks, our proposed model offers a more intuitive insight. Social science professionals and psychologists can swiftly understand the model’s rationale by merely contrasting code values. Consequently, our integrated model streamlines the interpretation of local model behaviors, bypassing the intrinsic nuances of TabNet’s inherent explanatory features. This enhancement widens its applicability to a more diverse audience.

A significant benefit of TabNet’s architecture is its ability to obviate the necessity for feature pre-processing. Through TabNet’s Mask layer, there is a streamlined selection of the most pivotal features (14). Such a mechanism bolsters the model’s efficiency, channeling its learning capability toward the most relevant features and circumventing unnecessary computations on superfluous ones. Hence, in our research, we trained the model directly without employing any feature selection techniques before model training. Yet, for conventional ML models to achieve optimal performance, the incorporation of an adept feature selection method is essential. Future studies might delve into the integration of tree-based models with several feature selection methods, subsequently contrasting their efficacy against our proposed SSP-TabNet model.

While some similar studies (2, 13, 43, 44), which applied ML models to predict life satisfaction, have reported slightly higher performance scores, it is crucial to acknowledge that these models often rely on extensive feature engineering or manual selection of relevant variables. In contrast, the SSP-TabNet model automatically identifies the most relevant features through its Mask layer. In our work, we seeks to illustrate that the standard architecture of our proposed SSP-TabNet is more robust than the standard architectures of tree-based ML models, which often require experimentation with various manual feature selection methods to achieve optimal performance. Additionally, many higher-performing models in previous studies (2, 13, 43) tend to function as “black-box” models, offering limited interpretability. Our model, therefore, strikes a balance between predictive accuracy and interpretability, which is critical for real-world applications, particularly in social science and mental health research.

## 5 Limitations and future works

This study presents several limitations. Firstly, the process of training and fine-tuning the TabNet model was more time-intensive compared to traditional machine learning models. Secondly, the generalizability of our predictive models is limited due to the specific cultural and socioeconomic context of the dataset, which originates from South Korea and includes individuals aged 15 and above. As a result, applying these models to different populations and cultural contexts may not be straightforward. Third, by relying on static, cross-sectional data, the model may not capture the temporal and dynamic aspects of life satisfaction, potentially failing to account for inherent fluctuations and evolving circumstances over time. Lastly, the stability of LIME explanations can occasionally be affected by variations in sample selection or the criteria used for including local data points in the model.

In future research, we intend to test and potentially strengthen the robustness of our models by integrating data from a variety of nations, with the goal of developing a more globally applicable predictive model. We intend to expand our study to encompass various demographic and cultural situations, allowing for a comprehensive assessment of the generalizability of the findings across varied populations. Additionally, we intend to subject the LIME interpretations derived from our model to meticulous review and evaluation by experts in social science and psychology.

## 6 Conclusion

This study introduced the SSP-TabNet model, a synergy of the self-supervised pre-training TabNet model and the LIME interpretative methodology, tailored to predict life satisfaction among South Koreans. The performance of our advanced model surpassed conventional tree-based ML models, registering an AUC of 0.7778 for the training set and 0.7757 for the test set. Furthermore, our integrated model simplifies and clarifies the interpretation of local model actions, effectively navigating past the intricate nuances of TabNet’s standard explanatory mechanisms. This refinement paves the way for wider accessibility and understanding across diverse audiences.

## Data Availability

The original contributions presented in the study are included in the article/Supplementary material, further inquiries can be directed to the corresponding author.
