# Supplementary material for: A hybrid self-supervised model predicting life satisfaction in South Korea
Source: Front Public Health. 2024 Oct 17;12:1445864. doi: 10.3389/fpubh.2024.1445864 (PMC11524807; doi:10.3389/fpubh.2024.1445864)
Supplement: Supplementary file 1 [file Table_1.DOCX]

Supplementary Material

# Supplementary Figures and Tables

Table S1. Variable descriptions.

| **Variable** | **Description** | **Field Type** |
| --- | --- | --- |
| code3 | ﻿Economic activity status in the past week | Categorical: 1. Engaged in, 2. Did not engage in |
| code12 | Factors causing employment difficulties in Busan | Categorical: 1. Lack of opportunities for open recruitment exams, 2. Difficulty in acquiring job information, 3. Lack of opportunities for technical training for employment, 4. Lack of an industrial base to absorb the workforce, 5. Unfavorable treatment of graduates from local universities, 6. Lack of financial means and connections for employment, 7. General economic and social uncertainties, 8. Various discriminations such as gender bias and age restrictions, 9. Others |
| code13 | Adequacy of Job Opportunities within the Local Area | Categorical: 1. Very much, 2. Somewhat, 3. Average, 4. Not quite, 5. Not at all |
| code14 | Job Search Activity | Categorical: 1. Yes, 2. No |
| code17 | Factors Influencing Job Choice | Categorical: 1. Prestige/Fame, 2. Stability, 3. Income, 4. Aptitude/Interest, 5. Fulfillment/Self-achievement, 6. Working Environment, 7. Development/Prospects, 8. Working Hours, 9. Other, 10. Don't know |
| code20 | ﻿Support Policies for Employment Expansion | Categorical: 1. Vocational Training, 2. Entrepreneurship Support, 3. Job Placement, 4. Job Creation, 5. Business Attraction and Employment Promotion, 6. Other |
| code21 | Satisfaction with Employment Expansion Policies | Categorical: 1. Very much, 2. Somewhat, 3. Average, 4. Not quite, 5. Not at all, 6. Uncertain |
| code22 | Total period of residence in Busan | Numeric: ( ) years |
| code23 | Duration of residence in current residential district | Numeric: ( ) years |
| code24 | Intention to settle in Busan | Categorical: 1. Very much, 2. Somewhat, 3. Average, 4. Not quite, 5. Not at all |
| code25 | Intention to Settle in the district or county | Categorical: 1. Very much, 2. Somewhat, 3. Average, 4. Not quite, 5. Not at all |
| code26 | Residential Environment Satisfaction: Housing | Categorical: 1. Very much, 2. Somewhat, 3. Average, 4. Not quite, 5. Not at all |
| code27 | Residential Environment Satisfaction: Infrastructure | Categorical: 1. Very much, 2. Somewhat, 3. Average, 4. Not quite, 5. Not at all |
| code28 | Residential Environment Satisfaction: Parking lot | Categorical: 1. Very much, 2. Somewhat, 3. Average, 4. Not quite, 5. Not at all |
| code29 | Residential Environment Satisfaction: Surrounding Walkability | Categorical: 1. Very much, 2. Somewhat, 3. Average, 4. Not quite, 5. Not at all |
| code30 | Public Transportation Satisfaction: Local Bus Service | Categorical: 1. Very much, 2. Somewhat, 3. Average, 4. Not quite, 5. Not at all, 6. Avoid using |
| code31 | Public Transportation Satisfaction: Intercity Bus Service | Categorical: 1. Very much, 2. Somewhat, 3. Average, 4. Not quite, 5. Not at all, 6. Avoid using |
| code32 | Public Transportation Satisfaction: Taxi Service | Categorical: 1. Very much, 2. Somewhat, 3. Average, 4. Not quite, 5. Not at all, 6. Avoid using |
| code33 | Public Transportation Satisfaction: Subway/ Light Rail Service | Categorical: 1. Very much, 2. Somewhat, 3. Average, 4. Not quite, 5. Not at all, 6. Avoid using |
| code34 | Do you commute or go to school? | Categorical: 1. Commute, 2. Go to school, 3. Do not commute or go to school |
| code39 | Satisfaction with Cultural and Leisure Facilities | Categorical: 1. Very much, 2. Somewhat, 3. Average, 4. Not quite, 5. Not at all |
| code41 | Satisfaction with Leisure Activities | Categorical: 1. Very much, 2. Somewhat, 3. Average, 4. Not quite, 5. Not at all |
| code43 | Time pressure in daily life on weekdays | Categorical: 1. Always, 2. Sometimes, 3. Almost never, 4. Never |
| code44 | Time pressure in daily life on weekend | Categorical: 1. Always, 2. Sometimes, 3. Almost never, 4. Never |
| code45 | Weekend and Holiday Leisure Activities | Categorical: 1. Watching TV, 2. Viewing culture and arts, 3. Participation in culture and arts, 4. Watch sports (go directly to the stadium and watch), 5. Sports activities, 6. Tourism activities, 7. Computer Games, Internet Browsing, etc., 8. Hobbies/self-development activities, 9. Relaxation activities (sleeping, sauna, etc.), 10. Social and other activities (volunteer activities, religious activities, social activities, etc.), 11. Others |
| code48 | Whether to watch the performance | Categorical: 1. Yes, 2. No |
| code57 | Effects of School Education: Acquisition of Knowledge and Skills | Categorical: 1. Very much, 2. Somewhat, 3. Average, 4. Not quite, 5. Not at all, 6. Uncertain |
| code58 | Effects of School Education: Personality Development | Categorical: 1. Very much, 2. Somewhat, 3. Average, 4. Not quite, 5. Not at all, 6. Uncertain |
| code59 | Effects of School Education: Formation of National and Social Perspectives | Categorical: 1. Very much, 2. Somewhat, 3. Average, 4. Not quite, 5. Not at all, 6. Uncertain |
| code60 | Effects of School Education: Utilization in Daily Life and Employment | Categorical: 1. Very much, 2. Somewhat, 3. Average, 4. Not quite, 5. Not at all, 6. Uncertain |
| code76 | Mainly Used Market | Categorical: 1. Traditional Market, 2. Large Supermarket, 3. Supermarket, 4. Neighborhood Convenience Store, 5. Sales corner in department store, 6. Online Shopping Mall, 7. Other |
| code77 | Measures to Revitalize Traditional Markets | Categorical: 1. Construction and Expansion of Parking Lots, 2. Modernization of Traditional Market Facilities, 3. Street Improvement, 4. Improvement in Transaction Methods (e.g., accepting credit card payments), 5. Enhancement of Customer Convenience Facilities, 6. Specialization of Businesses and Development of Unique Products, 7. Collaborative Events, Festivals, Promotions, etc., 8. Merchant Courtesy and Innovation Training, 9. Other |
| code109 | Gender | Categorical: 1. Male, 2. Female |
| code110 | Age | Numeric: ( ) years-old |
| code111 | Relationship with the Head of Household | Categorical: 1. Head of Household, 2. Spouse, 3. Unmarried Child, 4. Married Child and Their Spouse, 5. Grandchild and Their Spouse, 6. Parent (Including Spouse's Parents), 7. Grandparent (Including Spouse's Grandparents), 8. Unmarried Sibling (Including Siblings of the Spouse), 9. Other Relative, 10. Other housemates |
| code112 | Education Level | Categorical: 0. No Education, 1. Elementary School, 2. Middle School, 3. High School, 4. College (Less than 4 years), 5. University (4 years or more), 6. Master’s degree, 7. Ph.D.’s Degree |
| code114 | Marital Status | Categorical: 1. Single, 2. Married, 3. Divorced, 4. Widowed |
| code119 | Changes in Daily Life Due to COVID-19: Sleep Duration | Categorical: 1. Very much, 2. Somewhat, 3. Unchanged, 4. Not quite, 5. Not at all, 6. Not applicable |
| code120 | Changes in Daily Life Due to COVID-19: Employment activities | Categorical: 1. Very much, 2. Somewhat, 3. Unchanged, 4. Not quite, 5. Not at all, 6. Not applicable |
| code121 | Changes in Daily Life Due to COVID-19: Caring for children | Categorical: 1. Very much, 2. Somewhat, 3. Unchanged, 4. Not quite, 5. Not at all, 6. Not applicable |
| code122 | Changes in Daily Life Due to COVID-19: Housework activities | Categorical: 1. Very much, 2. Somewhat, 3. Unchanged, 4. Not quite, 5. Not at all, 6. Not applicable |
| code123 | Changes in Daily Life Due to COVID-19: Use of digital devices | Categorical: 1. Very much, 2. Somewhat, 3. Unchanged, 4. Not quite, 5. Not at all, 6. Not applicable |
| code124 | Changes in Daily Life Due to COVID-19: Leisure activities | Categorical: 1. Very much, 2. Somewhat, 3. Unchanged, 4. Not quite, 5. Not at all, 6. Not applicable |
| code125 | Changes in Daily Life Due to COVID-19: Religious activities | Categorical: 1. Very much, 2. Somewhat, 3. Unchanged, 4. Not quite, 5. Not at all, 6. Not applicable |
| code126 | Changes in Daily Life Due to COVID-19: Gatherings with Family, Friends, and Colleagues | Categorical: 1. Very much, 2. Somewhat, 3. Unchanged, 4. Not quite, 5. Not at all, 6. Not applicable |
| code127 | Behavior Changes for COVID-19 Prevention: Avoiding Going Out | Categorical: 1. Always, 2. Often, 3. Occasionally, 4. Never |
| code128 | Behavior Changes for COVID-19 Prevention: Cancelling Gatherings and Not Attending Events | Categorical: 1. Always, 2. Often, 3. Occasionally, 4. Never |
| code129 | Behavior Changes for COVID-19 Prevention: Avoiding Public Transportation | Categorical: 1. Always, 2. Often, 3. Occasionally, 4. Never |
| code130 | Behavior Changes for COVID-19 Prevention: Wearing a Mask | Categorical: 1. Always, 2. Often, 3. Occasionally, 4. Never |
| code131 | Behavior Changes for COVID-19 Prevention: Using Soap and Hand Sanitizer | Categorical: 1. Always, 2. Often, 3. Occasionally, 4. Never |
| code132 | Behavior Changes for COVID-19 Prevention: Indoor Ventilation | Categorical: 1. Always, 2. Often, 3. Occasionally, 4. Never |
